# Method for Patterning of Conductive Polymers on Flexible Substrates with Possible Applications for Wearable Sensing

**DOI:** 10.3390/mi17040467

**Published:** 2026-04-12

**Authors:** Mariya Aleksandrova, Georgi Nikolov, Valentin Mateev, Rade Tomov, Ivo Iliev

**Affiliations:** 1 Department of Microelectronics, Technical University of Sofia, 1756 Sofia, Bulgaria; rtomov@tu-sofia.bg; 2 Department of Electronics, Technical University of Sofia, 1756 Sofia, Bulgaria; gnikolov@tu-sofia.bg (G.N.); izi@tu-sofia.bg (I.I.); 3 Department of Electrical Apparatus, Technical University of Sofia, 1756 Sofia, Bulgaria; vmateev@tu-sofia.bg

**Keywords:** conductive polymers patterning, PEDOT:PSS, flexible substrates, wearable sensors

## Abstract

This study presents a novel fabrication approach for the precise patterning of conductive polymer coatings (graphene/PEDOT:PSS) on flexible substrates. Traditional lithographic methods often result in chemical or thermal degradation of polymer chains, compromising electrical conductivity. The proposed method utilizes an inversely structured gold nanocoating (400–450 nm) as a sacrificial template. By employing a selective lift-off process in a potassium iodide solution, high-resolution polymer topologies are achieved without damaging the active material. The resulting structures exhibit a sheet resistance of 90–100 Ω/sq and maintain linear sensitivity to temperature and humidity, making them suitable for next-generation wearable medical diagnostics.

## 1. Introduction

Conductive polymers are a type of polymeric material that possesses the ability to conduct electric current [1,2]. They are types of organic semiconductors that can be used to create flexible and biocompatible electronic devices and differ from ordinary non-conjugated polymers, which are typically insulators. Their “soft” organic nature makes them naturally flexible, unlike rigid inorganic semiconductors. Conductive or conjugated polymers can be used for various applications, including the fabrication of components such as sensors, displays, solar cells, and batteries [3,4,5,6]. They are necessary when implementing flexible devices to replace thin metal layers, which are brittle and crack easily under cyclic mechanical loading [7]. Their biocompatibility allows them to safely interface with living cells and fluids while converting biological events into direct electrical signals [8,9].

PEDOT:PSS (poly(3,4-ethylenedioxythiophene) and poly(styrenesulfonate)) is one of the most widely used conducting polymers in various applications, including biomedical electronics [10]. It is stable in air and moisture, which is essential for long-term applications in medical devices. It is often employed for making electrodes in devices like neural interfaces and cardiovascular sensors, where reliable electrical contact with biological tissues is essential [11,12,13,14,15]. PEDOT:PSS can be used in devices that respond to physiological signals, such as pressure or temperature, which can be beneficial for personalized medicine [16].

Miniature structures of conductive polymers with high resolution are important for sensors because they ensure precise localization of the active regions of the sensor, improving the detection of small changes in physical or chemical parameters [17,18,19]. Furthermore, miniaturization allows for the production of more compact sensors that can be integrated into various devices and applications, including wearable technologies—for example, sensors for various biomarkers in real-time, thanks to being continuously attached to the body [20]. The temperature gradient and the degree of skin perspiration can provide important information about an individual’s health state. Increased or decreased skin temperature can indicate inflammatory processes, infections, vascular problems, or even neurological disorders. For example, higher temperatures in certain areas may indicate a localized infection [21,22,23]. Perspiration is an important function for thermoregulation. Irregular or excessive hyperhidrosis (sweating) can be a symptom of various diseases, including endocrine disorders or neurological problems. The combination of temperature gradient and perspiration level can provide additional information. For instance, if skin temperature is elevated in combination with increased sweating, this could be an indicator of fever. Conversely, low temperature with decreased sweating may indicate a state of shock or circulatory problems [24,25,26]. Therefore, monitoring the temperature gradient and the degree of sweating is an important element of clinical diagnostics and can help identify underlying diseases or pathological conditions. This necessitates the use of flexible polymer substrates that are sensitive to processing with organic solvents, which wash away areas of photosensitive materials during patterning.

There are several microelectronic technologies that can provide high-resolution material patterning, which, however, prove to be inapplicable for conductive polymers. A well-known patterning method is optical (standard) lithography, which uses UV light to illuminate a photosensitive material (photoresist), which can then be processed to obtain a mask, protecting areas of the target layer for patterning from chemical dissolution (etching). The unprotected (not covered by photoresist) areas are etched, but conductive polymers do not have this property, as the process damages or destroys the polymer chain, resulting in a loss of conductivity [27]. Electron lithography is another conventional patterning approach in microelectronics that uses electron beams instead of focused light, allowing for even higher spot resolution and precision in the structures. However, it thermally deforms the polymer structure and, in addition, its cost is high [28]. Methods for patterning with specific geometry and small sizes of conductive polymer segments for precise sensor applications can involve printing in the desired shape with screen printing, inkjet printing, or ultrasonic atomization of solutions or inks of conductive polymers while observing the type of solvent and its compatibility with the flexible substrate [18,29]. A patterning method specifically developed for PEDOT:PSS films is achieved through selective oxygen plasma etching with a copper protection layer, enabling a minimum feature size of 20 µm. This patterned PEDOT:PSS film is effectively used as source–drain electrodes in organic field effect transistors and as a transparent anode in organic photodiodes, outperforming conventional indium–zinc oxide (IZO) anode [30]. Some authors discuss various patterning strategies for fabricating high-resolution PEDOT and PEDOT:PSS thin-film electrode arrays, including inkjet printing and laser patterning [31]. The minimum attainable size for patterns created by inkjet printing with oxidants is 50 μm. Laser ablation has patterned pure PEDOT electrodes with resolutions down to 1 μm, and a laser-induced phase separation method can achieve a resolution of 6 μm; however, this chemical composition, lacking PSS doping, is not as conductive as expected from an electrode. In [32], an electrochemical gelation (“electrogelation”) method for rapidly patterning PEDOT:PSS hydrogels on any conductive template, including curved and 3D surfaces, is presented. The ability to produce PEDOT:PSS hydrogels with minimum feature sizes down to 100 µm using the electrogelation patterning technique is demonstrated [32]. Photopatternable PEDOT: PSS hydrogels for photolithography have also been developed, and the authors state that the optimized direct photolithography process enables patterning down to 5 µm directly on flexible substrates [33]. A paper, reporting submicron resolution of approximately 450 nm achieved by nanoimprint lithography, highlights a limitation of shape—a PEDOT:PSS layer featuring uniform nanostructures, specifically arrayed as hemispheres with a 150 nm diameter and a 450 nm pitch, can only be fabricated [34].

In all of the above-mentioned cases, the methods suffer from insufficient resolution due to the mechanism of particle transport to the patterning site (the substrate surface). As a result of the processes, rounded edges of the topological features that do not have clearly defined geometry and dimensions are obtained [35]. The practical implementation of the known patterning methods is limited in terms of resolution (~1 μm is the minimum size that can be reproduced, with an accuracy of >0.1 μm). The objective of this work is to create a patterned coating of conductive polymer with resolution comparable to standard lithography, using a method that overcomes the limitations found in existing technologies (optical lithography, electron-beam lithography, and printing) in terms of resolution, compatibility with flexible substrates, and preservation of the electrical conductivity of the polymeric material. The proposed method provides a low-cost approach using conventional microfabrication technology, ensuring compatibility with flexible substrates. In addition, there is no limitation on feature shapes. The method can be successfully adapted to any kind of conjugated polymer, which extends its applicability for a broad range of electronic devices in the bioelectronics field.

## 2. Materials and Methods

**Figure 1:**
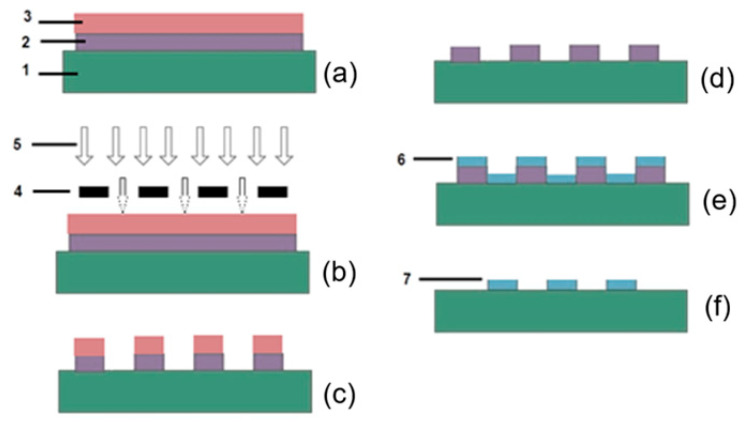
Technological flow for patterning the conductive polymer: 1—carrier (substrate); 2—gold coating; 3—photoresist; 4—photomask with inverse topology (negative); 5—ultraviolet light source; 6—conductive polymer; 7—graphene/PEDOT:PSS, patterned in the desired polymer topology.

Flexible polyethylene naphthalate (PEN) substrates (Goodfellow Advanced Materials, Huntingdon, Cambridgeshire, UK) were cleaned with isopropanol in a supersonic bath. Figure 1 presents the technological flow for patterning the conductive polymer. The essence of the method is in the use of a supplementary layer (called “sacrificial”) that is inversely patterned with respect to the desired topology, representing a gold nanocoating. First, a gold nanocoating with a thickness of 400–450 nm was deposited by vacuum sputtering of a gold sputtering target (Kurt J. Lesker Company, Dresden, Germany). It was coated with a positive tone liquid photoresist ma-P1215 (Micro Resist Technology GmbH, Berlin, Germany) (Figure 1a). The as-prepared system was subjected to a direct photolithographic patterning (Figure 1b), and the intermediate configuration shown in Figure 1c was obtained. The residual photoresist was stripped (Figure 1d). Next, a solution of a polymer composite graphene/PEDOT:PSS (Merck, Darmstadt, Germany) with a concentration of 0.2 mg/mL for PEDOT:PSS and 1 mg/mL for electrochemically exfoliated graphene was sprayed onto the newly formed topographic pattern, forming a layer with a thickness half that of the gold coating (Figure 1e). A Siansonic spray coater (Beijing, China) was used, and the spraying conditions were a substrate temperature of 110 °C, solution flow rates of 5 mL/min, and carrier gas flow rates of 2 psi. The thickness of the polymer layer was maintained at half the thickness of the gold layer to prevent the covering of the vertical sidewalls, ensuring a clean lift-off. Following this, the gold coating was treated in an aqueous solution of potassium iodide with a component ratio of 1:4:40 (I_2_/KI/H_2_O). This process intentionally and non-aggressively dissolves the gold on the flexible substrate at a low rate, leading to the lift-off effect but avoiding interface tearing (Figure 1f). It means that segments of the gold coating, along with the conductive polymer coating, are washed away. However, the segments deposited directly onto the substrate remained unaffected because the conductive polymers do not dissolve in the inorganic solvent (potassium iodide in this case). As a result, the patterning of the conductive polymer was completed, yielding a topology that exactly matches the intended design. The thickness of the conductive polymer layer was adjusted to achieve a sheet resistance of 90 to 100 Ω/square, which is low enough for it to serve effectively as an alternative electrode.

**Figure 2:**
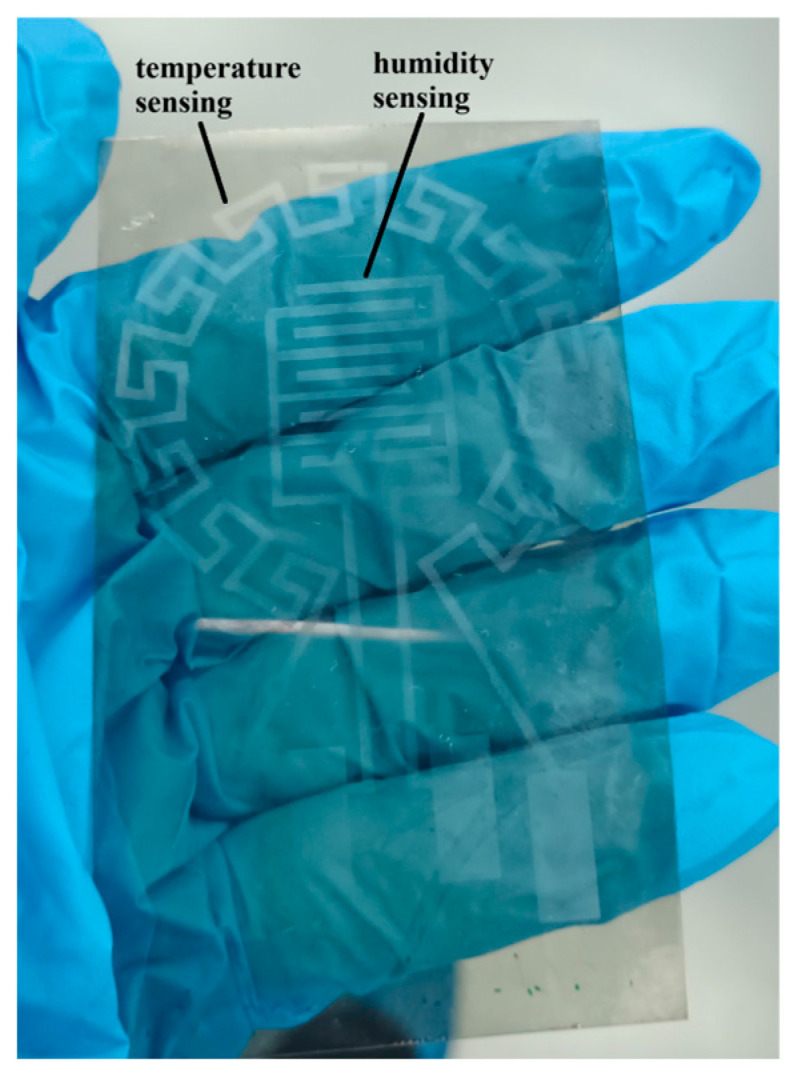
Photo of the combined sensing structures.

The coating is patterned in the form of a branched thermoelectric transducer combined with opposing comb-shaped (interdigitated—IDT) electrodes coated with a moisture-sensitive polymer polyamide (PA) solution (Paris, France) for capacitive moisture measurement (Figure 2). The resulting layer can be placed in direct contact with the skin without causing irritation, as it is biocompatible [36,37,38]. The outer contour is shaped like a rosette for precise temperature sensing. This specific geometry allows the sensor to capture thermal changes from multiple directions, providing more accurate measurements than standard designs. It is also related to the enhanced spatial resolution, which helps in understanding how temperature is distributed across a surface and offers versatility by accommodating different mounting orientations or surface shapes on the body. The interdigitated (IDT) pattern of the humidity sensing part is a standard, and it was used because it is highly sensitive to moisture changes, offers fast response times, and allows for miniaturization, making it easy to nest within the larger temperature rosette. The exact design of the sensing parts was previously established by the authors and reported elsewhere [39,40]. The core contribution of this work lies in the fabrication methodology, rather than the design optimization of the patterns. Beyond the introduction of a novel fabrication technique, the focus of this study lies in the characterization of the resulting graphene/PEDOT:PSS structures through rigorous performance testing. To validate the efficacy of the sacrificial gold template method, the sensors were subjected to environmental conditions that significantly exceed the typical physiological range of human skin, including temperatures up to 70 °C and humidity levels exceeding 65%. This intentional testing under extreme parameters, coupled with extensive mechanical fatigue analysis up to 10,000 bending cycles, serves to establish the ultimate operational limits and structural robustness of the conductive polymer patterns, ensuring their reliability for high-performance wearable diagnostics.

The resistance change of the patterned graphene/PEDOT:PSS films with temperature was measured in the temperature range from 35 °C to 70 °C with the Van der Pauw method, realized by a Veeco FPP 5000 four-point probe (Veeco Instruments Inc., San Jose, CA, USA). The measurements were conducted using a Peltier element TEC1-12706 (Hebei I.T., Shanghai, China) as a heater. For the humidity structure testing, a chamber with a controlled environment was used, which accommodated the measured device and commercial KTJ/TA 318 hygrometer (Bonajay Technology, Shenzhen, China) with readings in percentage, indicating the amount of water vapor present in the air. A moisture-absorbing coating of polyamide (PA) was spray-coated on the top of the interdigitated electrodes of the humidity sensing part. The capacitance change with moisture in the range was measured by a Hioki IM3590 impedance analyzer (Nagano, Japan) in the frequency range from 1 Hz to 200 kHz. The samples were subjected to atomic force microscopy (AFM) analysis (FlexAFM, Nanosurf, Liestal, Switzerland) to measure the coating uniformity, average roughness, coverage integrity, and defects after lift-off.

The specifications of the used materials and instrumentation are as follows. The tensile modulus of the PEN sheets was 5 GPa; the purity grade of the gold target was 99.99%; the photoresist spectral sensitivity was 365 nm and the exposure dose at this wavelength was 45 mJ/cm^2^; the dispersion solvent of the graphene/PEDOT:PSS solution was dimethylformamide (DMF) with a boiling point of 105 °C at a pressure of 7 Torr (933.256 Pa); the nozzle of the spray-coater was a ConeMist type, generating droplets with sizes less than 15 µm; the temperature of pre-use processing of the PI was 120 °C; the accuracy of the four-point probe was ±0.5%; the power consumption of the Peltier module was 60 W at a temperature difference of 70 °C; the operating humidity range of the hygrometer was 10% RH to 90% RH; and the base accuracy of the impedance analyzer was ± 0.05% in the frequency range from 1 mHz to 200 kHz.

## 3. Results and Discussion

**Figure 3:**
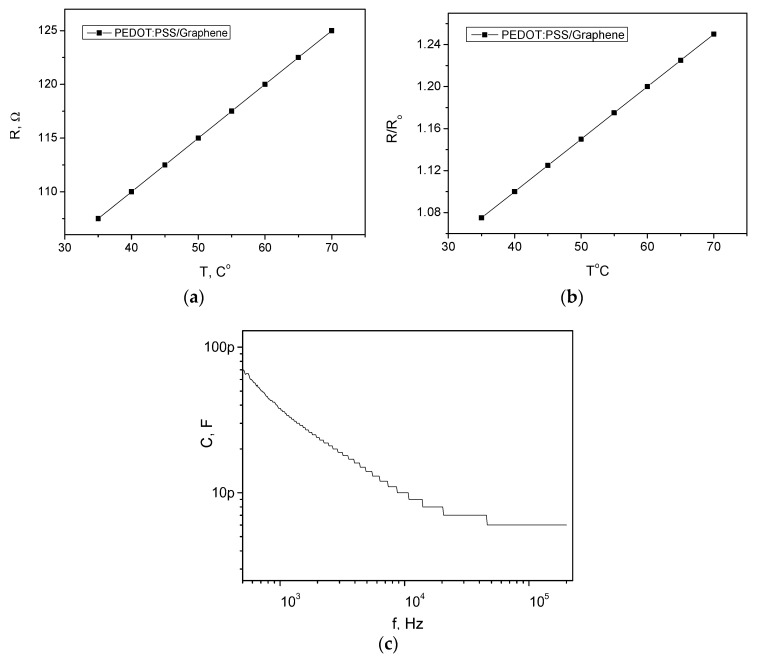
Electrical characterization of the sensing structures fabricated by lift-off graphene/PEDOT:PSS: (**a**) Resistance vs. temperature variation of the temperature sensing rosette; (**b**) relative resistance change at different temperatures from the measured range; (**c**) capacitance–frequency characteristic at humidity levels above 65%.

This section provides validation of the proposed sacrificial gold template method, establishing a direct correlation between the structural integrity of the polymer and its functional performance. The electrical characterization of the graphene/PEDOT:PSS patterns reveals a sheet resistance of 90–100 Ω/sq, which confirms that the selective lift-off process in potassium iodide solution effectively preserves the conjugated chain of the polymer. Unlike traditional lithographic methods that cause chemical or thermal degradation, this approach maintains high electrical conductivity, as evidenced by the linear relationship between resistance and temperature shown in Figure 3a,b. This linearity indicates a stable positive temperature coefficient, which is a prerequisite for reliable calibration in wearable medical diagnostics.

**Figure 4:**
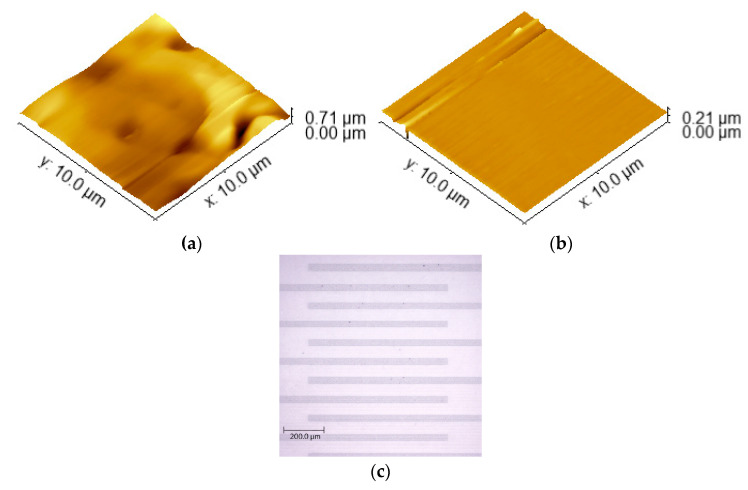
Three-dimensional AFM images of the (**a**) patterned by lift-off of the graphene/PEDOT:PSS coating of the PEN substrate and (**b**) PEN substrate surface. (**c**) Microscopic image of sample interdigitated electrodes fabricated by the proposed technology flow.

The morphological analysis conducted via AFM serves as structural proof for the efficacy of the fabrication process. As illustrated in Figure 4a, the conductive segments exhibit a high degree of uniformity and smoothness, with no evidence of the interface tearing that typically limits the resolution of printing (Figure 4b presents data on the uniformity and smoothness of the substrate for comparison). This topology is logically interconnected with the sensor’s sensitivity; the precise localization of active regions ensures that small fluctuations in physiological parameters are accurately captured. To demonstrate the ability of this method to eliminate rounded edges and poorly defined dimensions, a microscopic image of interdigitated electrodes with a scale of 200 µm is shown in Figure 4c. Furthermore, the IDT moisture-sensing structure within the temperature-sensing rosette, as depicted in Figure 2, demonstrates a functional synergy. The moisture sensor relies on the variation of the dielectric constant within the polyamide coating, a mechanism that remains uncompromised by the patterning process, allowing for simultaneous monitoring of skin temperature and perspiration. In Figure 3c, the capacitance–frequency characteristic is presented for the interdigitated (IDT) moisture sensor after the graphene/PEDOT:PSS was patterned using the sacrificial gold template method. It displays the capacitance–frequency characteristic at humidity levels above 65%, which is typical for high humidity conditions in such a sensor. This information is significant, as it validates the functional synergy of the IDT electrodes within the temperature rosette. Moreover, the structure effectively forms a planar microcapacitor with a typical capacitance vs. frequency curve. Figure 3c demonstrates that the device maintains proper capacitive behavior across a broad frequency range, confirming its structural robustness and suitability for the accurate capacitive principle of measuring humidity, despite the replacement of the metal electrodes and their patterning by a non-lithography process. If the conductive polymer edges are poorly defined or the lift-off is incomplete, parasitic resistance is introduced. The device behaves as an RC network rather than a capacitor. The impedance analyzer will not distinguish between changes in humidity (dielectric change) and changes in the polymer’s resistance (ohmic change), leading to noisy data. In addition, a true microcapacitor relies on a uniform electric field passing through the moisture-sensitive layer. If the lift-off process results in rounded edges or inconsistent spacing between the IDT fingers, the electric field becomes non-homogeneous. The sensitivity becomes non-linear, and the ability to use a standard calibration curve is lost, because different areas of the sensor respond differently to the same moisture level.

Since the fabrication method relies on selective chemical etching (using a potassium iodide solution), the presence of residual gold is theoretically eliminated by the nature of the process. Because the gold is chemically removed, it cannot influence the electrochemical stability or biocompatibility of the final sensor. The device interfaces with the skin using only the “soft” organic polymer, which is inherently biocompatible and does not cause irritation. To ensure a clean lift-off of the gold under the polymer, the thickness of the polymer layer was maintained at exactly half the thickness of the gold layer. This prevents the polymer from covering the vertical sidewalls of the gold pattern. It ensures the etchant has direct access to the gold interface, allowing for complete delamination of the sacrificial segments. The chemical selectivity of the iodine-based etchant is a fundamental advantage of this fabrication method. Iodine from the etching solution components serves to convert gold into gold iodide, while the potassium iodide provides excess iodide ions to solubilize the gold iodide into an aqueous complex. The etching time was precisely estimated based on industry standards for this concentration, because for the used ratio at room temperature, the typical etch rate is approximately 0.5 to 1.0 µm/minute (according to the agitation conditions and freshness of delivery of the etching fluid). To ensure 100% removal of a 450 nm layer, the immersion time should be approximately 30 to 60 s. In practice, a slight over-etch of 90 s was used to account for the “shadowing” effect of the polymer edges and to guarantee a clean lift-off at the interface. The atomic force microscopy (AFM) analysis in the study (Figure 4b) serves as physical proof of this cleanliness. If the selective chemical etching process were incomplete, residual gold would appear as “islands” or clusters, instead of the flat profile of the PEN. An AFM scan would show sharp vertical spikes in this case.

**Figure 5:**
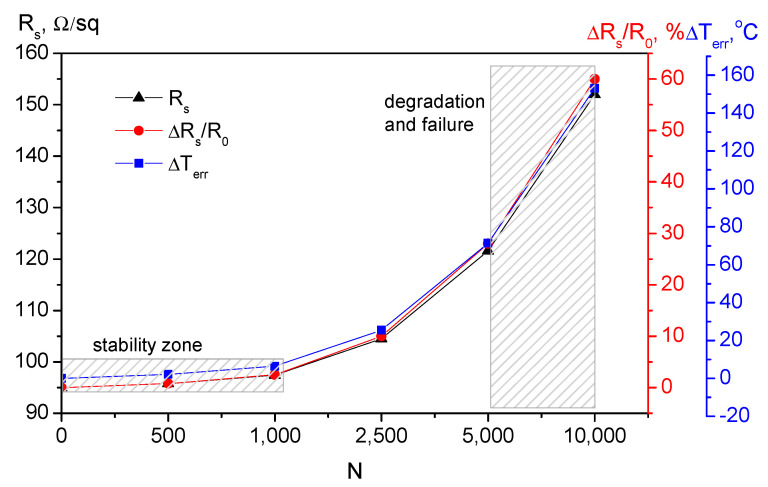
Stability data for the graphene/PEDOT:PSS lifted-off patterns of the temperature sensor and sensor performance at multiple bending cycles.

**Figure 6:**
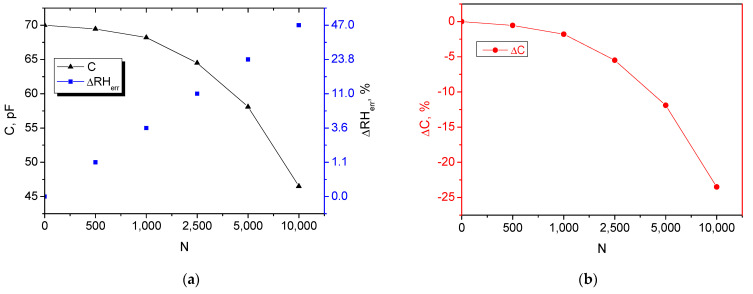
Stability data for the moisture absorbing/graphene/PEDOT:PSS lifted-off patterns of the humidity sensor. (**a**) Capacitance variation and sensor error at multiple bending cycles. (**b**) Capacitance deviation affecting the sensor performance.

Investigating the relationship between resistance and the number of bending cycles (N) is critical for developing reliable flexible electronics. A resistive temperature sensor works by measuring small changes in resistance caused by heat. However, mechanical bending also changes resistance by creating micro-fractures. Flexible sensors are often integrated into wearable tech (smart clothing) or robotics (artificial skin). These applications involve constant movement and require the fatigue limit to be defined. The same assumptions are valid for the humidity sensor. Table 1 summarizes the deviation of the resistance from its initial value because of the bending and the induced measurement error, as visualized in Figure 5. The same approach was applied for the humidity sensor, and the results are summarized in Table 2 and visualized in Figure 6. The drop from 70 pF downwards indicates that the electrodes are losing their effective area due to stress.

The sheet resistance increased from 95 to 152 Ω/sq at 10,000 cycles, which can be caused by the formation of micro-fractures within the graphene/PEDOT:PSS polymer chain due to repeated stress. Because the sensor detects heat via small resistance changes, these structural damages are misinterpreted as a massive temperature measurement error of 153.1%. The capacitance drops from 70.00 pF to 46.50 pF at the same number of bending cycles. This reduction probably occurs because mechanical stress causes the electrodes to lose their effective surface area, directly hindering the sensor’s ability to store charge and resulting in a misinterpretation as a false decrease in humidity levels, or a humidity error of −47%.

The protocol for the bending cycling of flexible structures with conductive polymer coatings (non-patterned) was developed earlier by our group and reported elsewhere [41]. Following the same principle, the bending tests were conducted at 10,000 cycles that were chosen to push the device beyond normal use to establish its absolute operational limits and structural robustness. The data in the tables show a progression where high stability is maintained up to 1000 cycles (the slope is shallow), but deviations in the measured quantities occur at 2500 cycles. By 10,000 cycles, the device reaches critical failure (for humidity measurement) or device failure (for temperature measurement). In the zone defined as stable, the temperature sensor maintains a relatively constant sheet resistance, which only increases by 0.8%, resulting in a minimal induced temperature error of 2.1 °C and humidity error of 3.6%. By 2500 cycles, a structural fatigue is suggested (the slope increases sharply), as the the temperature error (Δ*T_err_*) significantly increases to 25 °C and the humidity error (Δ*RH_err_*)—to 11% RH, and by 10,000 cycles, the device reaches critical failure, where the induced temperature error is 153.1 °C and the humidity error is −47%. When bending causes mechanical damage, the resulting increase in resistance is misinterpreted by the system as a temperature change. The error is calculated as:

(1) $${∆T}_{err}=f\left(\frac{{\Delta R}_{s}}{R_{0}}\right)$$

for the temperature measurements, where *R*_0_ is the initial resistance before the applied bending, Δ*R_s_* is the variation of the sheet resistance.

(2) $${∆RH}_{err}\sim f\left(\frac{1}{∆C}\right)\;for\;the\;humidity\;measurements,where\;\Delta C\;is\;the\;variaction\;of\;capacitance.$$

The experimental results of this study demonstrate that the proposed sacrificial gold template method successfully overcomes the limitations related to other patterning technologies while maintaining the polymer’s intrinsic electrical properties. Table 3 compares the performance and constraints of the developed approach against existing patterning methodologies reported in the literature.

**Remark** **1.** *In microelectronics, the resolution for patterning is not a single fixed value but is defined by the specific technological generation (node) and application. It is defined as the minimum half-pitch size. High-resolution patterns must maintain non-rounded edges and low roughness (typically < 20% of the feature size) to be functional, which is proven by AFM.*

## 4. Conclusions

The findings of this study demonstrate that the preservation of the material’s “soft” organic nature through a non-aggressive sacrificial gold template method directly translates into superior sensor performance. By utilizing an inversely structured gold nanocoating and a selective lift-off process, high-resolution graphene/PEDOT:PSS topologies were achieved without the chemical or thermal degradation typical of traditional lithographic methods. The high initial mechanical stability observed is attributed to the fact that the coating’s structural integrity and adhesion remain entirely unaffected during this non-aggressive patterning, preventing the pre-existing micro-defects often introduced by laser or thermal methods. Electrical characterization confirmed this integrity, yielding a consistent sheet resistance of 90–100 Ω/sq and a stable, linear relationship between resistance and temperature. Furthermore, the integration of an interdigitated moisture-sensing structure enabled precise capacitive humidity measurements, remaining uncompromised by the patterning process. By overcoming the resolution and degradation limitations of existing technologies, this scalable, low-cost fabrication pathway provides a robust method for integrating complex, multifunctional conductive polymer circuits onto flexible substrates for next-generation personalized healthcare monitoring. It should be noted that the primary focus of this study is the validation of the sacrificial gold template patterning methodology rather than the exhaustive characterization of the humidity sensor’s long-term drift or response dynamics. The environmental and mechanical stress tests performed (up to 70 °C and 10,000 bending cycles) serve to demonstrate that the proposed fabrication process effectively preserves the structural and electrical integrity of the conductive polymer. While the sensing performance is consistent with the properties of the active materials used, the stability of the patterned structures confirms that this method is a robust and viable alternative to traditional lithography for high-resolution wearable electronics.

The method is not limited to one material and can be adopted for any kind of conjugated polymer to replace metal electrodes in wearable sensor devices. Because it employs standardized microelectronic fabrication technologies, it is applicable to a broad range of sensor elements measuring various non-electrical quantities related to the assessment of patients’ health.

## Figures and Tables

**Table 1:** Resistance degradation over bending cycles at room temperature and with a bending radius of 10 mm.

Bending Cycles, N	Sheet Resistance, Rs, Ω/sq	Resistance Change, ΔR_s_/R_0_, %	Induced Temp. Error, ΔT_err_, °C	Sensor Status
0	95.0	0.0	0.0	Initial state
500	95.8	0.8	2.1	High stability
1000	97.4	2.5	6.4	Early drift
2500	104.5	10.0	25.5	Structural fatigue
5000	121.6	28.0	71.4	Critical degradation
10,000	152.0	60.0	153.1	Device failure

**Table 2:** Capacitance degradation over bending cycles at room temperature and with a bending radius of 10 mm.

Bending Cycles, N	Capacitance, C, pF	Capacitance Deviation, ΔC, %	Induced Humidity Error, ΔRH_err_, %	Sensor Status
0	70.00	0.00	0.0	Initial state
500	69.45	−0.55	1.1	Minimal Drift
1000	68.20	−1.80	3.6	Material Fatigue
2500	64.50	−5.50	11.0	Visible Deviation
5000	58.10	−11.90	23.8	Major Structural Damage
10,000	46.50	−23.50	47.0	Critical Failure

**Table 3:** Comparison of patterning methods for conductive polymers and polymer composites.

Method	Resolution	Advantages	Key Disadvantages	Ref. No.
**Proposed method**	High (~400 nm)	Preserves conductivity; no feature shape limits.	Requires vacuum sputtering of gold.	**This Work**
**Optical lithography**	High (lower than 400 nm)	Standard microfabrication technology.	Chemical/thermal degradation of polymer chains.	[27]
**Electron-beam lithography**	Very high (lower than 10 nm)	Exceptional precision and spot resolution.	High cost; thermal deformation of the polymer.	[28]
**Inkjet printing**	Very low (~50 µm)	Low cost; direct deposition on substrates.	Rounded edges; insufficient resolution.	[18]
**Laser ablation**	Low (1–6 µm)	High resolution for pure PEDOT.	Lacks PSS doping; lower conductivity.	[31]
**Nanoimprint lithography**	High (~400 nm)	Submicron resolution achieved.	Limited to specific shapes (e.g., hemispheres).	[34]

## Data Availability

The original contributions presented in this study are included in the article. Further inquiries can be directed to the corresponding author.

## Abbreviations

The following abbreviations are used in this manuscript:

PEDOT:PSS	Poly(3,4-ethylenedioxythiophene):poly(styrenesulfonate)
PEDOT	Poly(3,4-ethylenedioxythiophene)
PEN	Polyethylene naphthalate
IDT	Interdigitated
AFM	Atomic force microscopy
